# Advances in SRNS Gene Research: From Precision Classification to Precision Diagnosis and Treatment

**DOI:** 10.3390/biomedicines14030711

**Published:** 2026-03-19

**Authors:** Yuhong Ye, Limin Huang, Haidong Fu, Jingjing Wang, Yanyan Jin

**Affiliations:** Department of Nephrology, Children’s Hospital, Zhejiang University School of Medicine, National Clinical Research Center for Children and Adolescents’ Health and Diseases, Hangzhou 310052, China

**Keywords:** steroid-resistant nephrotic syndrome (SRNS), genetic classification, precision diagnosis, gene therapy, podocytes, glomerular filtration barrier

## Abstract

To clarify the genetic classification, diagnostic strategies, and precision treatment pathways of steroid-resistant nephrotic syndrome (SRNS), this review systematically reviews the genetic stratification system of SRNS by integrating recent advances in genetic testing technologies and pathogenesis research. It contains the pathogenic mechanisms, diagnostic protocols, and therapeutic correlations of different genetic subtypes, while summarizing current progress and clinical challenges in gene therapy. Results indicate SRNS can be categorized into genetic (38–58%) and non-genetic/immune-mediated (40–60%). A stepwise diagnostic system comprising core proteinuria gene panel testing, whole-genome sequencing (WGS), whole-exome sequencing (WES), and supplementary multi-omics/long-range sequencing is proposed, suited for populations with “typical phenotypes and moderate genetic risk”, “atypical phenotypes and high genetic suspicion”, and “complex structural/non-coding region variants” respectively. Pathogenic mechanisms directly determine therapeutic strategies: *COQ2/PDSS2* mutations respond to coenzyme Q10 suplementation, while *NPHS1* mutations necessitate early renal transplantation. Adeno-associated virus (AAV)-mediated gene therapy and CRISPR-Cas editing show preclinical promise but face challenges including incomplete detection coverage and clinical translation difficulties. Genetic technologies are driving SRNS management transformation from “empirical treatment” to “mechanism-oriented precision diagnosis and therapy”. Future efforts should focus on overcoming genetic testing limitations and gene therapy translation bottlenecks to enhance diagnostic and therapeutic efficacy.

## 1. Introduction

Nephrotic syndrome (NS) represents the primary category of glomerular diseases in children, with a global incidence ranging from 1.15 to 16.9 cases per 100,000 population. Among these cases, steroid-resistant nephrotic syndrome (SRNS) accounts for 15–20% [1,2]. SRNS exhibits a high propensity to progress to end-stage renal disease (ESRD), with a 5-year ESRD incidence reaching 30–50%. Owing to the fact that traditional immunosuppressive therapies yield a response rate of less than 20%, SRNS has emerged as a significant diagnostic and therapeutic challenge in the field of pediatric nephrology [2,3].

In recent years, with the widespread adoption of next-generation sequencing (NGS) technology and the proposal of the “two-hit” theory, which posits that the pathogenesis of SRNS requires the synergistic action of the first hit (inherited genetic susceptibility, such as monogenic/polygenic variants or genetic background that impairs glomerular filtration barrier function) and the second hit (acquired environmental or immune triggers, including infection, inflammation, and drug exposure) [4,5], etiological research on SRNS has advanced from a “phenotype association” approach to a “gene-mechanism” level. To date, over 70 monogenic disease-causing genes for SRNS have been identified, encompassing multiple functional pathways such as podocyte cytoskeleton, glomerular basement membrane, and energy metabolism [5,6,7]. Precision diagnosis and targeted therapy based on genetic typing can significantly reduce drug side effects resulting from ineffective treatments and improve patient prognosis [8]. This article systematically describes the genetic stratification system, stepwise diagnostic strategy, mechanism-oriented therapeutic regimens, and advances in gene therapy for SRNS, aiming to provide a reference for clinical practice and academic research.

## 2. Precision Classification of SRNS: Stratification Based on Genetics and Pathogenesis

Advancements in genetic testing technologies and mechanistic research have transformed the classification of steroid-resistant nephrotic syndrome (SRNS) from an “empiric phenotypic classification” to a “mechanism-oriented precision classification,” enabling more targeted therapeutic strategies. With the popularization of the “two-hit” hypothesis and genetic testing, SRNS classification now centers on the “genetic vs. non-genetic (immune-mediated)” axis, directly guiding individualized treatment. The specific categories are as follows.

### 2.1. Genetic SRNS (Accounting for 38–58% of Pediatric SRNS Cases)

Genetic SRNS takes monogenic hereditary forms as the core pathogenic subtype (30–50% of pediatric SRNS cases), with digenic/polygenic hereditary variations (approximately 8%) serving as modifier factors for the main genetic cause rather than an independent etiological subtype. Digenic/polygenic variations typically act in synergy with a major monogenic pathogenic mutation, leading to increased phenotypic severity but no distinct clinical phenotype independent of the core monogenic subtype, and the overall case number is limited with significant phenotypic overlap with monogenic forms.

Monogenic hereditary SRNS is characterized by the absence of obvious immune-inflammatory evidence in most cases (rather than absolute absence), with partial cases presenting with non-specific mild abnormalities in individual inflammation markers; serum complement levels are mostly normal, and autoantibodies are absent in the majority of cases [9]. Genetic testing allows definitive diagnosis, thereby avoiding ineffective immunosuppressive therapy [10]. This subtype is caused by pathogenic mutations in single genes and can be further subdivided based on gene function, with distinct therapeutic regimens and prognoses corresponding to different subtypes, as shown in Table 1:

Digenic/polygenic genetic variations in SRNS are defined as the synergistic effect of a major pathogenic monogenic mutation combined with one or more additional genetic modifier factors, which are associated with faster disease progression and poorer prognosis compared with single monogenic mutations, but do not form an independent clinical subtype. Common mutation combination patterns include the following categories (redefined as modifier combination patterns) [11,12], as shown in Table 2:

### 2.2. Non-Genetic/Immune-Mediated SRNS (40–60%)

Lacking definite genetic pathogenic drivers, this subtype involves dysregulated immunity targeting podocytes, serving as the main non-genetic subtype of SRNS and corresponding to the original immune-mediated subtype definition, as shown in Table 3:

### 2.3. Genetic–Immunological Interactions in SRNS

SRNS represents a complex disorder driven by bidirectional genetic–immune regulation, involving direct genetic effects on immunity, immune modulation of gene expression, and critical mediation by cross-regulatory genes, all influenced by environmental factors.

**Figure 1:**
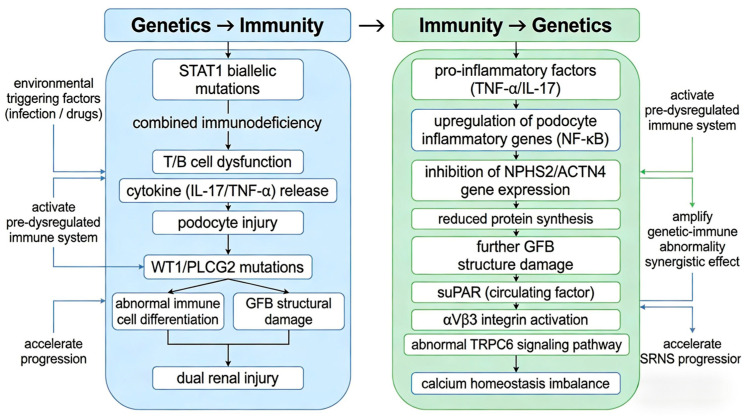
Schematic diagram of the molecular pathway of SRNS-mediated genetic—immune bidirectional regulation.

Molecular mechanisms involve bidirectional pathways; the bidirectional regulatory network between genetic mutations and immune dysregulation is shown in Figure 1, with cross-regulatory genes and environmental factors acting as key mediators. Genetic mutations can directly impair immune function, as exemplified by biallelic STAT1 mutations causing combined immunodeficiency with subsequent podocyte injury, forming a “genetic defect—immune dysregulation—renal lesion” cascade [13]. Conversely, immune dysregulation reciprocally modulates gene expression—proinflammatory cytokines like TNF-α disrupt podocyte cytoskeletal integrity, while Th17 cell-derived IL-17 upregulates podocyte inflammatory genes, exacerbating injury [14,15,16]. Critical cross-regulatory genes bridge renal structure and immune function. WT1 maintains glomerular architecture while regulating immune cell differentiation, and PLCG2 stabilizes the glomerular filtration barrier while transducing immune signals. Their aberrations simultaneously disrupt genetic stability and immune homeostasis [17,18].

Genetic susceptibility renders kidneys vulnerable to immune-mediated damage. Environmental triggers (infection, medications) activate pre-dysregulated immune systems, with combined genetic—immune abnormalities accelerating progression [19,20]. Clinically, concurrent genetic variants and immune dysfunction synergize to worsen renal impairment in many patients [21].

SRNS exhibits non-specific clinical presentations, complicating subtype classification [22]. Treatment response heterogeneity—ranging from refractoriness to partial remission—likely stems from interactions between individual genetic backgrounds, immune dysregulation profiles, and environmental factors [23,24,25].

## 3. Genetic Diagnosis of SRNS: Adaptation Table for Different Populations

The core technologies of genetic diagnosis for SRNS are designed to balance “precision” and “cost-effectiveness,” clarifying the application boundaries of three mainstream detection methods to form a complementary and synergistic stepwise diagnostic system. These methods include core proteinuria gene panel testing, whole-genome sequencing (WGS) [26], whole-exome sequencing (WES), and supplementary multi-omics/long-range sequencing, each tailored to distinct patient populations based on clinical and genetic characteristics.

### 3.1. Core Gene Panel Testing: First-Line Screening for “Typical Phenotype, Moderate Genetic Risk” Populations

As the first-line screening tool, the core proteinuria gene panel covers over 40 pathogenic genes, encompassing more than 90% of known disease-causing loci. It is optimally suited for patients with the following features:

1. Children with isolated symptoms: Presenting with classic nephrotic syndrome without extrarenal complications and showing no response to steroid therapy.
2. Mid-age onset Children: Onset between 1 and 12 years (a period where variations in core pathogenic genes like *NPHS2* and *ADCK4* account for 62% of cases, with stable phenotypes and no extreme age-related rare genetic features).
3. Sporadic cases: No family history of kidney disease, non-consanguineous parents, and no family members with early-onset renal failure or similar nephropathy. Pathogenic genes typically involve high-frequency common variants (e.g., *NPHS2* gene *R138Q* mutation) [7,27,28].

### 3.2. Whole-Exome Sequencing (WES): Upgraded Protocol for Negative Core Panel Results, Suited for “Atypical Phenotype, High Genetic Suspicions” Populations

WES serves as a supplementary testing when core proteinuria gene panel results are negative but clinical suspicion of genetic etiology remains high. It is indicated for populations with atypical phenotypes and strong genetic associations, characterized by:

1. Complex cases with extrarenal symptoms: Beyond nephrotic syndrome, concurrent extrarenal abnormalities such as hearing/visual impairment (suggesting *COL4A5*-related Alport syndrome); ocular structural defects (e.g., congenital cataracts, warranting *LAMB2* gene screening); or neuromuscular symptoms (e.g., muscle weakness, potentially linked to *INF2* gene variants).
2. Extreme age at onset: Onset < 1 year (infantile SRNS, where monogenic causes account for 61%, often involving rare variants poorly covered by core panels); onset > 12 years (adolescent/adult SRNS, with lower genetic contribution but frequently involving genes like *ACTN4* and *TRPC6* not included in core panels).
3. Definite familial genetic background: First-degree relatives (parents, siblings) with nephrotic syndrome or chronic renal failure; consanguineous parents (offspring at higher risk of carrying homozygous rare variants not covered by core panels); or multiple affected family members (suggesting autosomal dominant inheritance, e.g., *TRPC6* dominant mutations) [29,30,31].

### 3.3. Whole-Genome Sequencing (WGS): Core Upgraded Detection for Refractory Cases with Structural/Non-Coding Variant Suspicions

WGS serves as the core upgraded testing for genetic diagnosis of SRNS, targeted at refractory cases with negative core proteinuria gene panel results and suspected non-coding region variants, chromosomal structural abnormalities, or extremely rare genetic backgrounds [32]. It is the preferred upgraded detection strategy after first-line screening, capable of directly detecting deep intronic variants and regulatory region abnormalities that cannot be covered by WES, and is adapted for the following populations:

1. Suspected non-coding region variants: Clinically strong genetic suspicion (e.g., clear family history, typical phenotype) but negative WES for coding region variants, requiring investigation of non-coding anomalies such as gene promoter methylation defects (e.g., *NPHS2* promoter abnormalities affecting gene expression) or deep intronic variants causing mRNA splicing errors—a typical type of loss-of-function variant (e.g., large insertions in *WT1* introns).
2. Suspected chromosomal structural abnormalities: Karyotype analysis or chromosomal microarray (CMA) indicating segmental abnormalities (e.g., large deletions/duplications); suspected pathogenic chromosomal translocations or inversions (e.g., large deletions in the 11p13 region harboring *WT1*); or multi-gene joint variants requiring analysis of non-coding regulatory networks beyond WES coverage.
3. Globally rare refractory cases: Negative results from both panel and WES testing with no known pathogenic genes matching the phenotype; enrollment in rare disease research programs to identify novel causative genes (e.g., non-coding RNA or regulator variants); or cases requiring genetic exclusion to guide treatment after inconclusive multidisciplinary consultations [7,33].

### 3.4. Multi-Omics and Long-Range Sequencing: Supplementary and Exploratory Detection for Refractory Negative Cases

This section focuses on two emerging technologies that are currently in the research and exploration stage and have not been routinely used in clinical practice, serving as important supplementary and exploratory means for refractory SRNS cases with negative results of conventional sequencing technologies (core proteinuria gene panel/WGS/WES). The two technologies target different types of rare mutations and structural variations, forming a complementary relationship with conventional detection methods.

#### 3.4.1. Multi-Omics Technology: Supplementary Detection for Rare Mutations with Functional Abnormality Suspicions

Multi-omics technology acts as a supplementary detection method for rare SRNS mutations, integrating genomic, transcriptomic, proteomic and metabolomic data to analyze pathogenic mutations from multiple dimensions of gene sequence, expression and function. It makes up for the deficiency of single gene sequencing that only focuses on sequence variation and cannot confirm the functional abnormality of mutant genes.

It is mainly applicable to refractory cases with negative conventional sequencing results and suspected rare mutations with functional abnormalities, such as low-abundance chimeric mutations, gene expression regulation abnormalities caused by epigenetic modifications, and pathogenic mutations with normal sequences but abnormal protein translation. At present, this technology is rarely used in clinical practice and is mainly applied in basic research and individualized diagnosis of rare and refractory SRNS. With the improvement of detection technology and the reduction of cost, it is expected to realize clinical transformation and become an important supplementary means of genetic diagnosis [33,34,35].

#### 3.4.2. Long-Range Sequencing: Exploratory Detection for Conventional Short-Read Sequencing Negative Cases

Long-range sequencing is an important exploratory detection technology for SRNS cases with negative results of all conventional short-read sequencing technologies (core proteinuria gene panel/WGS/WES). It breaks the splicing limitations of traditional short-read sequencing and can accurately detect complex structural variations that are difficult to identify by short-read sequencing, such as large fragment insertions/deletions, chromosomal inversions, tandem repeats, and complex translocations. These complex structural variations are important pathogenic factors of SRNS that are easily missed by conventional detection methods, and their accurate detection is of great significance for clarifying the etiology of refractory cases [33,36].

This technology is currently in the clinical exploration stage for genetic diagnosis, and its application is limited by high technical costs and lack of unified clinical testing standards [37]. With the continuous optimization of sequencing technology, the reduction of detection costs and the formulation of standardized operating procedures, long-range sequencing is expected to become a routine test for SRNS genetic diagnosis in the near future and be integrated into the stepwise diagnostic system to solve the detection dilemma of complex structural variations.

## 4. Clinical Diagnostic System for SRNS: Gene-Directed Therapy and Monitoring

### 4.1. Mechanistic Differences Among Genes and Therapeutic Implications

Monogenic genetic defects account for approximately 30–50% of SRNS etiologies, primarily involving genes related to the structure and function of the glomerular filtration barrier, particularly podocytes. The molecular mechanistic differences among various gene mutations directly determine the selection of distinct therapeutic strategies [8,38]. This section elaborates on three key aspects: gene classification, mechanistic differences, and therapeutic correlations.

#### 4.1.1. Classification and Functional Background of SRNS-Associated Core Genes

**Figure 2:**
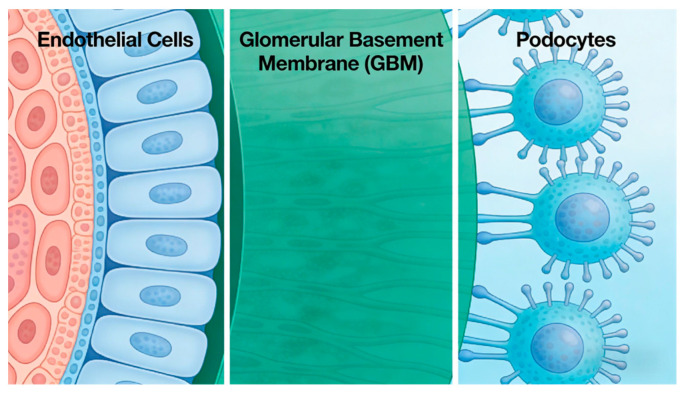
Structure of the glomerular filtration barrier (GFB).

The glomerular filtration barrier consists of three layers: “endothelial cells–basement membrane–podocytes” (shown in Figure 2). The structural integrity of podocytes (glomerular visceral epithelial cells) is critical for maintaining filtration function—podocytes form interdigitating connections via “foot processes,” and the “slit diaphragm (SD)” between adjacent foot processes serves as the final barrier preventing protein leakage [39,40]. SRNS-associated genes primarily regulate four core podocyte functions, slit diaphragm assembly, cytoskeletal stability, signaling pathway regulation, and energy metabolism, and can be specifically categorized into four classes.

Slit diaphragm structural genes directly constitute the slit diaphragm and maintain filtration barrier integrity. Podocyte cytoskeletal genes, including actin filaments and actin-binding proteins, stabilize foot process morphology and regulate cellular contraction and adhesion. Signaling pathway regulatory genes indirectly influence filtration barrier function by controlling podocyte differentiation, survival, or basement membrane synthesis. Energy metabolism genes sustain mitochondrial function to ensure podocyte energy supply [41,42]. These functional categories collectively orchestrate podocyte homeostasis, with pathogenic variants in any class disrupting the delicate balance required for normal glomerular filtration.

#### 4.1.2. Differential Pathogenic Mechanisms Among Genes

**Figure 3:**
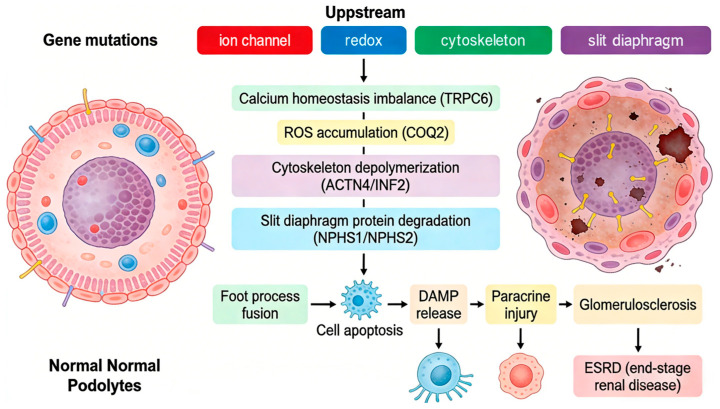
Core molecular cascade of podocyte injury leading to progressive renal pathology.

Despite the different upstream genetic triggers, the podocyte injury induced by SRNS pathogenic mutations converges to a common molecular cascade reaction, which is the core basis of SRNS progression (Figure 3). Genetic defects cause SRNS by disrupting distinct podocyte functions, with core genes categorized into four functional classes based on their mechanistic roles:

##### Slit Diaphragm Structural Disruption Type (e.g., *NPHS1*, *NPHS2*)

Nephrin, encoded by the *NPHS1* gene, represents the core component of the slit diaphragm. It forms “filtration pores” through extracellular domain homodimerization, while its intracellular domain interacts with signaling molecules to regulate podocyte survival [43]. Mutations in *NPHS1* cause Nephrin deficiency or dysfunction, representing a primary etiology of congenital nephrotic syndrome [44].

Podocin, a membrane-associated slit diaphragm protein encoded by *NPHS2* [45,46,47], stabilizes Nephrin localization and function through direct binding, serving as the primary protein facilitating Nephrin translocation to the slit diaphragm. *NPHS2* mutations induce Podocin conformational abnormalities that disrupt Nephrin interaction, reducing slit diaphragm stability and triggering podocyte foot process degeneration—another established cause of congenital nephrotic syndrome.

##### Podocyte Cytoskeleton Disorder Subtype (e.g., *ACTN4*, *INF2*, *TRPC6*)

*ACTN4* is a protein that maintains podocyte foot process structural stability to withstand intraglomerular filtration pressure while accommodating dynamic pressure changes through subtle conformational adjustments—functions critical for crosslinking actin filaments and regulating cytoskeletal architecture [48,49,50]. *ACTN4* mutations predominantly occur within the actin-binding domain, a key site for actin filament interaction. These mutations enhance protein polymerization capacity, impair podocyte motility, prevent adaptation to filtration pressure, and progressively induce podocyte apoptosis.

*INF2* acts as a master regulator of actin filament assembly and disassembly, sustaining the flexibility and stability of foot process cytoskeletons [51]. Most *INF2* mutations exhibit dominant inheritance, with pathogenic mechanisms centered on two core pathways: “cytoskeletal disorganization” and “stress-induced injury.” Mutant *INF2* triggers excessive actin filament polymerization, causing irreversible foot process contraction and fusion. Aberrantly polymerized actin filaments not only disrupt structural integrity but also initiate secondary damage through “cytoskeleton–organelle crosstalk,” leading to massive podocyte death. Apoptotic podocytes further release damage-associated molecular patterns that amplify podocyte injury cascades [52,53].

*TRPC6* encodes a nonselective cation channel on podocyte membranes that modulates intracellular Ca^2+^ concentrations. By mediating controlled Ca^2+^ influx and regulating phosphorylation of actin-binding proteins, *TRPC6* maintains the elasticity and stability of foot process cytoskeletons [54]. *TRPC6* mutations induce persistent channel activation, resulting in intracellular Ca^2+^ overload. This activates calcium-dependent proteases, ultimately degrading cytoskeletal proteins, causing foot process disintegration, and triggering podocyte death [55].

##### Signal Pathway Abnormality Type (e.g., *WT1*, *LAMB2*, *CD2AP*)

*WT1* is a transcription factor that maintains podocyte homeostasis through three primary mechanisms: regulating podocyte-specific gene expression, preserving podocyte differentiation and maturation, and participating in the regulation of glomerular basement membrane (GBM) synthesis [56]. WT1 mutations induce podocyte dysfunction through three core pathways: “transcriptional regulation failure,” “differentiation impairment,” and “multi-pathway abnormal activation” [45]. The specific mechanisms are as follows: 1. Loss of *WT1* transcriptional activity, leading to dysregulated target gene expression. 2. Arrest of podocyte differentiation and maturation defects. 3. Aberrant activation of multiple injury pathways, accelerating podocyte death [56,57,58].

*LAMB2* is a key member of the laminin family, which together with LAMA5 and LAMC1 forms the GBM-specific LN-521 complex—a core component maintaining GBM structural integrity and signaling functions. It acts primarily by constructing the GBM structural scaffold, mediating adhesion signals between podocytes and GBM, and regulating podocyte differentiation and maturation [59]. Over 80 pathogenic *LAMB2* mutations have been identified, with missense and nonsense mutations being the most common. Disease pathogenesis involves three core processes: “GBM structural disruption,” “adhesion signal loss,” and “podocyte functional collapse.” The specific mechanisms are: 1. Impaired assembly of the LN-521 complex, resulting in GBM structural collapse. 2. Interrupted adhesion signaling, leading to imbalance in podocyte survival and cytoskeletal homeostasis. 3. Secondary inflammation and glomerulosclerosis [60,61].

*CD2AP* is a “linker molecule” between the slit diaphragm and podocyte cytoskeleton, serving as a key regulator of glomerular podocyte structure and function with dual core roles in maintaining renal filtration barrier integrity and cell survival [62]. *CD2AP* acts both as a “bridge molecule” to sustain the slit diaphragm structure and directly determines podocyte survival status by regulating apoptotic signaling pathways [51]. The “structural linkage” and “apoptosis regulation” functions of *CD2AP* are interdependent, with dysfunction in either triggering cascading effects. The specific mechanisms are: 1. Reduced binding of *CD2AP* to Nephrin/actin, causing slit diaphragm disintegration and impaired linkage function. 2. Decreased *CD2AP* expression, increased podocyte apoptosis, and failure of apoptotic regulation. 3. Congenital functional defects caused by *CD2AP* gene mutations [63,64].

##### Energy Metabolism Disorder Type (e.g., *COQ2*, *PDSS2*)

Podocytes are highly metabolically active and energy-dependent cells. Mitochondrial dysfunction directly leads to podocyte failure. These genetic defects belong to “coenzyme Q10 (CoQ10) synthesis disorders” [65]. Both *COQ2* and *PDSS2* are key enzymes for CoQ10 synthesis. CoQ10 serves as an electron carrier in the mitochondrial respiratory chain and maintains energy production [65]. The specific mechanism is as follows: gene mutations result in insufficient CoQ10 synthesis, impaired mitochondrial oxidative phosphorylation, and reduced ATP production, leading to podocyte apoptosis due to energy depletion; simultaneously, reactive oxygen species (ROS) accumulate, exacerbating cellular damage [66,67].

#### 4.1.3. Correlation Between Pathogenic Mechanism Differences and Therapeutic Strategies in SRNS

The core principle of SRNS treatment is to select targeted or symptomatic therapy based on the “mechanism type” of genetic defects, avoiding blind use of immunosuppressants (some genetic defects involve no immune abnormalities, rendering immunotherapy ineffective while increasing side effects). The specific correlations are shown in the following Table 4:

### 4.2. Precision Intervention in Monogenic Cases

#### 4.2.1. Significance of Precision Intervention

Statistics indicate that approximately 15–20% of nephrotic syndrome patients exhibit steroid resistance, among whom about 38–58% of SRNS cases are caused by genetic factors (30–50% core monogenic mutations + 8% monogenic mutations combined with genetic modifier factors) [6,68]. With the advancement of genetic technologies, more than 57 monogenic disease-causing genes (included in the UK GMS Proteinuria panel) with multiple pathogenic variants have now been identified to be associated with SRNS pathogenesis, most of which are related to podocyte function [2].

Precision intervention refers to the formulation of individualized treatment strategies based on a patient’s specific genotype, phenotype, and disease mechanism. In monogenic SRNS cases, precision intervention holds significant importance. Firstly, a definitive genetic diagnosis can avoid unnecessary immunosuppressive therapy. Studies have shown that patients with genetic SRNS have an extremely low response rate to traditional immunosuppressants, with only 3% achieving complete remission and 16% achieving partial remission [68]. Therefore, after genetic diagnosis, excessive use of immunosuppressive drugs should be strictly avoided to protect renal function and reduce drug side effects. Secondly, precision therapy targeting specific core monogenic mutations has shown significant efficacy in some cases. For example, in patients with mutations in CoQ10 synthesis-related genes (e.g., *COQ2*, *ADCK4*), exogenous supplementation of CoQ10 can achieve significant reduction or even complete remission of proteinuria [65]. Finally, precision intervention also includes genetic counseling and prenatal diagnosis. By clarifying the inheritance pattern of the core monogenic mutation and conducting carrier screening, the recurrence risk of the disease in families can be effectively reduced.

#### 4.2.2. Current Status of Precision Intervention

##### Intervention for Structural Protein-Related Genetic Defects (e.g., *NPHS1*, *NPHS2*, *ACTN4*)

Defects in slit diaphragm and cytoskeletal structural proteins directly impair the integrity of the glomerular filtration barrier.

Proteins encoded by *NPHS1* and *NPHS2* genes are core components of the slit diaphragm, and no direct replacement therapy currently exists. Primary intervention strategies include: 1. Supportive therapy: strict blood pressure control, use of RAS inhibitors to reduce proteinuria, protein intake restriction, and maintenance of nutritional status. 2. Avoidance of nephrotoxic drugs: avoidance of medications that may exacerbate renal injury, such as nonsteroidal anti-inflammatory drugs. 3. Renal replacement therapy: for patients progressing to end-stage renal disease, kidney transplantation is the primary treatment modality. Notably, the recurrence rate after renal transplantation is low in *NPHS1* mutation patients, while recurrence may occur in *NPHS2* mutation patients [69,70].

*ACTN4* is an actin-crosslinking protein. *ACTN4* gene mutations enhance its binding capacity to actin, leading to excessive crosslinking and stiffness of the cytoskeleton, inhibiting dynamic remodeling of podocyte foot processes, and subsequently causing podocyte injury [48,49,50]. Primary intervention strategies include: 1. RAS inhibitors: angiotensin-converting enzyme inhibitors or angiotensin II receptor antagonists can indirectly reduce excessive cytoskeletal load by lowering intraglomerular pressure and decreasing mechanical stretching of podocytes. 2. Low-protein diet combined with nutritional support: a protein intake of 0.8–1.0 g/kg standard body weight/day is recommended, with concurrent supplementation of α-keto acids to reduce renal metabolic load while preventing malnutrition. 3. Avoidance of nephrotoxic factors: strict prohibition of nephrotoxic drugs such as nonsteroidal anti-inflammatory drugs and aminoglycoside antibiotics; control of blood pressure and blood glucose (if diabetes is present) to reduce secondary podocyte injury. 4. Renal replacement therapy: kidney transplantation is the preferred option; hemodialysis or peritoneal dialysis may be selected before transplantation, requiring comprehensive preoperative evaluation to exclude transplantation contraindications [48,49].

##### Intervention for Signaling Pathway-Related Genetic Defects (e.g., *WT1*)

As a transcription factor, *WT1* gene mutations affect podocyte development and functional maintenance. Diseases caused by *WT1* mutations include Denys–Drash syndrome and Frasier syndrome [56]. Primary intervention strategies include: 1. Tumor surveillance: due to the risk of Wilms tumor, regular abdominal ultrasound examinations are required. 2. Hormone replacement therapy: hormone replacement therapy is necessary for patients with gonadal dysfunction. 3. Renal replacement therapy: most patients require kidney transplantation, with preoperative evaluation for malignant tumors [68,71].

##### Intervention for Energy Metabolism-Related Genetic Defects (e.g., CoQ10)

CoQ10 is a critical component of the mitochondrial respiratory chain. CoQ10 deficiency represents an important etiology of SRNS, leading to reduced ATP production and reactive oxygen species accumulation, ultimately causing podocyte dysfunction. Related genes include *PDSS1*, *PDSS2*, *COQ2*, and *COQ6*. For these genetic defects, exogenous CoQ10 supplementation can bypass the impaired biosynthetic pathway and restore mitochondrial function, which has now become the standard treatment [65].

### 4.3. Genetic Heterogeneity of SRNS

Genetic heterogeneity, a prevalent and critical feature in hereditary diseases, manifests distinctly and complexly in steroid-resistant nephrotic syndrome (SRNS), which is mainly divided into allelic heterogeneity and locus heterogeneity in molecular genetics. Allelic heterogeneity refers to the phenomenon that different variants of the same gene lead to the same or similar disease phenotypes; locus heterogeneity means that variants of different non-allelic genes cause the same clinical phenotype, both of which are the core manifestations of genetic heterogeneity in SRNS [72,73,74]. In contrast, phenotypic heterogeneity is a distinct genetic characteristic, which refers to the phenomenon that individuals with the same pathogenic genotype exhibit different clinical phenotype characteristics (including disease onset age, severity, progression rate, etc.) due to the influence of genetic background, environmental factors, epigenetic modification and other individual characteristics.

Taking *NPHS1*, a classic pathogenic gene in SRNS, as an example, this gene encodes Nephrin, a core protein of the glomerular slit diaphragm that plays an irreplaceable role in maintaining the integrity of the glomerular filtration barrier. When different types of variants occur in the *NPHS1* gene, different molecular and cellular effects are induced, and further lead to distinct clinical phenotypes, which is a typical performance of allelic heterogeneity. Variants that result in complete loss of protein function (e.g., nonsense variants, frameshift variants, splicing variants) can disrupt the normal expression or structure of Nephrin, leading to the complete loss of slit diaphragm function, and typically present as congenital nephrotic syndrome with onset before or within 3 months of birth, accompanied by severe symptoms such as massive proteinuria, hypoalbuminemia, and severe edema; the disease progresses rapidly and is prone to develop into end-stage renal disease without timely intervention. In contrast, variants that cause partial impairment of protein function (e.g., some missense variants) only reduce the expression or biological activity of Nephrin but do not completely abolish its function, and may delay the disease onset until childhood or even adulthood, with clinical phenotypes often manifesting as focal segmental glomerulosclerosis (FSGS); the symptoms are relatively mild, with lower levels of proteinuria, less severe edema and slower disease progression. It is worth noting that the clinical phenotype severity induced by the same type of *NPHS1* variant is not fixed; even for the same functional complete loss variant, there may be differences in onset age, symptom severity and progression rate among different patients, which is a typical performance of phenotypic heterogeneity. This phenomenon is closely related to individual characteristics such as the patient’s genetic background, epigenetic modification level, and environmental exposure, indicating that the clinical phenotype of SRNS is not only determined by the type of pathogenic variant but also regulated by multiple individual factors [75,76,77].

Locus heterogeneity is another important manifestation of genetic heterogeneity in SRNS, which is characterized by the fact that variants in different pathogenic genes lead to the same or highly similar clinical phenotypes of SRNS. Although these distinct pathogenic genes differ in chromosomal location, encoded protein products, and specific biological functions, they ultimately converge by disrupting critical components of the glomerular filtration barrier, triggering the typical clinical symptoms of SRNS and resulting in significant overlap in clinical phenotypes [78,79].

In SRNS, mutations in multiple genes including *NPHS1*, *NPHS2*, *WT1*, and *PLCE1* are common etiological factors, and patients with mutations in these genes all exhibit the core clinical symptoms of SRNS, including massive proteinuria (24 h urine protein >3.5g), hypoalbuminemia (serum albumin < 30g/L), generalized edema, and hyperlipidemia. Based solely on routine clinical symptoms, signs, and imaging findings, it is difficult to directly distinguish the specific pathogenic gene. The molecular basis for this phenotypic overlap lies in the fact that these distinct pathogenic genes all represent critical components of the podocyte functional regulatory network: Nephrin (encoded by *NPHS1*) and Podocin (encoded by *NPHS2*) are core structural proteins of the glomerular slit diaphragm, jointly maintaining the structural integrity of the filtration barrier; *WT1*, as a transcription factor, regulates the expression of genes involved in podocyte development and function. Regardless of their specific mechanisms, mutations in these genes ultimately cause podocyte dysfunction, disrupt the integrity of the glomerular filtration barrier, and trigger typical SRNS symptoms such as proteinuria, forming the phenotypic heterogeneity characteristics of “different genes, same phenotype” [80,81,82,83].

In summary, the genetic heterogeneity of SRNS is primarily manifested through two forms: allelic heterogeneity and phenotypic heterogeneity. Its essence results from the combined effects of mutation diversity in pathogenic genes and the complexity of gene functional networks. This heterogeneity not only reveals the diversified pathogenesis of SRNS but also holds important guiding significance for clinical practice: in diagnosis, due to phenotypic heterogeneity, clinical symptoms alone cannot identify the pathogenic gene, requiring integration of genetic testing for precise diagnosis; in treatment, different pathogenic genes and mutation types correlate with varying treatment responses and prognoses, making genotyping essential for personalized therapeutic regimens and improved treatment outcomes; in prognostic assessment, the differences in disease severity and progression rates between “severe” and “mild” mutations in allelic heterogeneity provide important references for predicting patient outcomes.

### 4.4. Advances in Gene Therapy for SRNS

Clinically, traditional drug therapy can only slow the progression of SRNS but cannot achieve a cure. Therefore, gene therapy represents one of the most promising treatment approaches for monogenic SRNS cases, with the following current advancements.

#### 4.4.1. Adeno-Associated Virus-Mediated Gene Therapy

In August 2023, the University of Bristol published a study in *Science Translational Medicine* demonstrating adeno-associated virus (AAV)-mediated gene therapy targeting mutations in the podocyte gene *NPHS2*. The AAV-LK03 serotype efficiently transduces human podocytes, and AAV-LK03-mediated Podocin transduction achieved functional rescue in mutant podocytes [84]. In mouse models, AAV 2/9-mediated gene transfer successfully ameliorated kidney disease; by incorporating small interfering RNA (siRNA) or short hairpin RNA (shRNA) into AAV vectors, RNA interference mechanisms enabled silencing of disease-causing genes [85,86].

Despite the enormous potential of AAV gene therapy in SRNS treatment, multiple challenges remain in translating laboratory research to clinical applications. These challenges can be summarized into four core dimensions: targeted delivery efficiency, immune barriers, balance between long-term efficacy and safety, and clinical translation bottlenecks.

Low targeted delivery efficiency is the primary challenge facing AAV-based SRNS therapy. The complex anatomical structure and hemodynamic characteristics of the kidney hinder effective enrichment of AAV vectors in cells associated with the glomerular filtration barrier [87]. Studies have shown that after intravenous administration, AAV vectors primarily accumulate in the liver with relatively little distribution to the kidney. Furthermore, the glomerular filtration barrier further limits vector penetration, particularly for large molecular weight vectors like AAV [88].

Immunological risks represent another critical challenge. SRNS patients may have pre-existing anti-AAV antibodies that can neutralize the vector and significantly reduce therapeutic efficacy [89]. Research indicates that approximately 30–60% of the population has anti-AAV antibodies, with significant variations in antibody levels across regions and age groups. Additionally, vector delivery may trigger de novo immune responses, including humoral and cellular immunity, which not only potentially reduce therapeutic efficacy but may also exacerbate kidney damage [90,91].

#### 4.4.2. Preclinical Advances and Translation Challenges with CRISPR-Cas

CRISPR-Cas applications primarily focus on preclinical research including cell lines, gene-edited organoids, and animal models. In urine-derived human podocyte cell lines, *COL4A5* mutations can be corrected, achieving a 40% mutation reversal rate in urine-derived podocyte lines from Alport syndrome patients. Using CRISPR base editing to generate human pluripotent stem cells differentiated into kidney organoids enables the establishment of disease models to investigate gene intervention targets, which holds great significance for understanding disease mechanisms and clinical trials. Another promising avenue involves using CRISPR technology to generate human kidneys in pigs, aiming to reduce the hyperoncogenicity of xenografts [92,93].

Despite the tremendous potential of CRISPR-Cas technology in SRNS treatment, translating laboratory research to clinical applications faces multiple challenges. First, inefficient kidney-targeted delivery: the complex renal anatomy, glomerular filtration barrier obstruction, and insufficient carrier specificity for renal cells hinder precise and efficient delivery of the CRISPR-Cas system to target cells such as glomerular podocytes and mesangial cells. Second, off-target effects and genomic safety risks: the CRISPR-Cas system may induce cleavage at unintended genomic loci, triggering gene mutations, chromosomal abnormalities, and potentially increasing cancer risk. Third, prominent immune-related risks: CRISPR-Cas proteins (mostly of bacterial origin) act as foreign antigens, potentially eliciting innate and adaptive immune responses that not only reduce editing efficiency but may exacerbate kidney injury. Fourth, clinical translation bottlenecks, including the lack of animal models accurately simulating human SRNS pathological features, inconsistent standards for quantifying and evaluating gene editing efficiency, and inadequate regulatory policies, all of which severely restrict the clinical translation of CRISPR-Cas technology in SRNS treatment [94,95].

## 5. Dilemmas and Prospects of Genetic Diagnosis and Treatment for SRNS

### 5.1. Dilemmas in Genetic Diagnosis and Treatment of SRNS

Current challenges in SRNS genetic diagnosis and treatment exhibit a prominent “bidirectional obstruction” characteristic, concentrated in the “incompleteness” of genetic testing and the “difficulty in translation” of gene therapy. These two factors mutually constrain each other, preventing the formation of an effective closed-loop in the entire diagnosis and treatment system and hindering the translation of genetic research findings into clinical benefits.

At the genetic testing level, the core issue is the mismatch between the limitations of testing scope and the complexity of pathogenic genes. Currently, over 70 disease-causing genes have been identified to be associated with SRNS pathogenesis, with continuous additions. These genes are widely distributed across multiple critical pathways such as renal development, glomerular filtration barrier construction, and immune regulation. However, existing routine clinical genetic testing methods, such as targeted gene capture sequencing and Sanger sequencing, mostly focus on known high-frequency core pathogenic monogenic genes (e.g., *NPHS1*, *NPHS2*, *WT1*). For genetic modifier factors (low-frequency mutations, rare genetic variants, non-coding region variations), copy number variations, chromosomal structural abnormalities, and unreported novel pathogenic genes, detection sensitivity is extremely low or completely absent. In addition, the complementary application of multiple detection technologies (core proteinuria gene panel/WGS/WES) is limited by the lack of standardized clinical protocols, leading to the underutilization of the respective advantages of each technology. For refractory cases with negative conventional short-read sequencing results, the application of long-range sequencing is limited by high technical costs and lack of clinical testing standards, leading to the inability to detect complex structural variations; multi-omics technology is rarely used in clinical practice due to the complexity of detection and data analysis, making it difficult to dig out rare mutations with functional abnormalities. This leaves many SRNS patients in a “cause-unknown” predicament—patients with typical clinical symptoms and pathological changes cannot have their pathogenic roots identified through existing tests, failing to guide treatment and severely impeding genetic counseling and prenatal diagnosis for patient families. More concerningly, some patients may have core monogenic mutations combined with genetic modifier factors or gene–environment interactions, which existing detection technologies struggle to accurately resolve, further exacerbating diagnostic difficulties.

At the gene therapy translation level, even when patients fortunately obtain a definitive genetic diagnosis through testing, they often face the awkward situation of “known cause but no cure.” Current SRNS gene therapy remains in preclinical research or early clinical trial stages, with extremely limited mature treatment protocols. Most diagnosed patients can only rely on traditional treatments such as high-dose glucocorticoids and immunosuppressants, which have limited efficacy for genetically caused SRNS and cause severe long-term side effects including infections, osteoporosis, and hepatorenal toxicity. For example, SRNS caused by *WT1* gene mutations—where *WT1* plays a critical role in renal development and glomerular function maintenance—results in severe glomerular filtration barrier damage, rapid disease progression, and frequent progression to end-stage renal disease. However, no targeted gene therapy drugs have been developed for such patients, who remain dependent on traditional immunosuppressive regimens, severely weakening the clinical value of genetic diagnosis. Furthermore, gene therapy faces multiple technical bottlenecks: lack of efficient and safe gene delivery vectors (existing vectors have issues with poor targeting, strong immunogenicity, and insufficient delivery efficiency), risks of off-target effects in gene editing, and difficulties in maintaining long-term stability of post-treatment gene expression. These factors collectively hinder the transition of gene therapy from laboratory to clinical application. Concurrently, high treatment costs, ethical controversies, and incomplete regulatory frameworks further increase barriers to clinical translation.

### 5.2. Prospects of Genetic Diagnosis and Treatment for SRNS

Despite current dilemmas, the rapid development of molecular biology and genetic engineering technologies—particularly advances in genetic testing and breakthroughs in gene therapy core technologies—are creating new opportunities in this field. These developments hold promise to gradually break existing bottlenecks, enabling the leap from “etiological diagnosis” to “precision treatment” and bringing revolutionary therapeutic hope to patients.

In genetic testing technology upgrades, continuous innovations in next-generation sequencing are gradually addressing the “incompleteness” issue. Clinical applications of whole-exome sequencing (WES) and whole-genome sequencing (WGS) are expanding rapidly. Compared to traditional targeted sequencing, these technologies comprehensively cover all human coding genes or even entire genomes, enabling precise detection of known pathogenic gene variations while identifying novel disease-causing genes, rare variants, and complex genetic interactions—significantly improving SRNS etiological diagnosis rates. Additionally, emerging technologies like single-cell sequencing and spatial transcriptome sequencing further analyze the gene expression profiles of different renal cell types, facilitating deeper understanding of SRNS pathogenesis and identifying new targets for genetic testing. Multi-omics technology will be gradually integrated into clinical genetic diagnosis, and the combination of genomic sequencing with transcriptome, proteome and metabolome data will realize the comprehensive analysis of “gene sequence variation-function abnormality”, significantly improving the detection rate of rare mutations in SRNS. Long-range sequencing technology will be continuously optimized, and its cost will be reduced, and it will gradually be integrated into the routine genetic diagnosis system of SRNS, effectively solving the detection dilemma of complex structural variations that cannot be identified by traditional short-read sequencing. Furthermore, integration of artificial intelligence and big data into genetic testing promises rapid analysis and accurate interpretation of massive genetic datasets, automatically identifying pathogenic variants and predicting disease progression risks—markedly enhancing diagnostic efficiency and accuracy for more precise personalized treatment planning.

In gene therapy technological breakthroughs, developments in cutting-edge technologies provide core support for precision treatment. Continuous optimization of CRISPR-Cas9 gene editing technology has significantly improved editing efficiency and specificity while reducing off-target risks, enabling “site-specific repair” of specific pathogenic genes. For instance, in SRNS caused by homozygous *NPHS2* mutations, CRISPR technology could precisely repair mutation sites to restore normal gene function, addressing the disease at its source. Significant progress has been made in developing novel AAV (adeno-associated virus) vectors; modifications to capsid proteins have greatly enhanced renal tissue targeting and delivery efficiency while reducing immunogenicity—ensuring safe and efficient gene delivery. Moreover, next-generation gene editing tools such as base editing and Prime Editing further expand therapeutic applications by precisely correcting various point mutations, insertions, and deletions, offering treatment possibilities for more SRNS subtypes.

In the future, as these technologies mature and undergo clinical translation, SRNS genetic diagnosis and treatment are expected to form a complete closed-loop of “precision diagnosis—targeted therapy—efficacy monitoring”: comprehensive and accurate pathogenic gene diagnosis via whole-genome sequencing combined with AI-based disease progression prediction; development of personalized gene therapies using optimized editing technologies and novel vectors for precise repair or replacement of pathogenic genes; and real-time therapeutic effect monitoring through liquid biopsies and gene expression analyses for timely treatment adjustments. Additionally, with decreasing gene therapy costs, improved regulatory frameworks, and expanded multicenter clinical trials, gene therapy is expected to gradually enter routine clinical practice—benefiting more SRNS patients, thoroughly transforming the current “easy diagnosis but difficult treatment” paradigm, and advancing SRNS treatment into an era of precision and potential cure.

## 6. Conclusions and Future Perspectives

This study systematically clarified the genetic classification, diagnostic strategies, therapeutic pathways, and technological advancements of SRNS.

In terms of disease classification, SRNS has shifted from traditional phenotype-based empirical stratification to a precise system centered on genetic versus non-genetic (immune-mediated) subtypes. Genetic forms account for 38–58% of pediatric SRNS, dominated by monogenic mutations (30–50%), with digenic/polygenic variants acting as modifiers (8%) that exacerbate phenotypic severity. Non-genetic/immune-mediated cases constitute 40–60%. These subtypes are not mutually exclusive: genetic defects and immune abnormalities interact bidirectionally, and key regulatory genes may disrupt renal function through both structural and immune pathways. Most monogenic SRNS lacks obvious immune inflammation, although mild non-specific changes may exist, requiring careful clinical interpretation.

For diagnosis, a stepwise, precision–economical protocol has been established, using a targeted proteinuria gene panel as first-tier testing, WGS as core upgraded detection, WES as supplementary testing, and multi-omics/long-range sequencing as exploratory tools. Flexible combination of these methods improves the etiological diagnostic yield and supports subsequent treatment selection.

Therapeutically, SRNS management has entered a mechanism-driven era. Distinct pathogenic mechanisms of genetic and immune-mediated subtypes determine treatment pathways, and early genetic diagnosis helps avoid unnecessary immunosuppression in monogenic SRNS, reducing adverse effects and improving prognosis. Preclinical gene therapy approaches, including AAV-mediated delivery and CRISPR-Cas gene editing, show curative potential for monogenic SRNS. However, challenges such as low renal targeting efficiency, off-target effects, immunogenicity, and lack of standardized translation limit clinical application.

Overall, genetic advances have driven SRNS from empirical management to mechanism-guided precision medicine. Current limitations include incomplete coverage of rare variants and non-coding regions, as well as unmet curative options for many genetically defined patients. Future research should expand diagnostic coverage, optimize AAV and CRISPR-Cas technologies, and integrate AI and big data to refine genetic analysis. A closed-loop precision system of diagnosis-targeted treatment–monitoring will further improve SRNS outcomes and enable individualized care.

## Figures and Tables

**Table 1:** Functional classification of monogenic SRNS genes with pathogenic mechanisms.

Functional Category	Representative Genes	Pathogenic Mechanism
Podocyte cytoskeleton/adhesion	*NPHS1* (nephrin), *NPHS2* (podocin), *ACTN4*	Disrupted cytoskeletal stability causing glomerular filtration barrier (GFB) leakage
Glomerular basement membrane	*LAMB2*, *COL4A3/A4/A5*	Structural abnormalities impairing GFB integrity
Energy metabolism	*COQ2*, *COQ8B*, *PDSS2*	Mitochondrial respiratory chain dysfunction; podocyte energy depletion
Ion channels	*TRPC6*, *CLCN5*	Calcium/chloride homeostasis imbalance leading to foot process fusion
Transcriptional regulation	*WT1*, *PAX2*	Abnormal podocyte development with extrarenal malformations

**Table 2:** Genetic modifier combination patterns and clinical features in SRNS.

Mutation Combination Category	Representative Mutation Combination	Core Clinical Features
Energy metabolism gene + Podocyte cytoskeleton gene	*COQ2* + *ACTN4*	Rapid disease progression with accelerated deterioration of renal function based on the core monogenic phenotype
Glomerular basement membrane (GBM) gene + Transcriptional regulatory gene	*LAMB2* + *WT1*	Severe extrarenal manifestations (cataracts, genital malformations) superimposed on the core monogenic renal phenotype
Non-coding region variant + Coding region mutation	*NPHS2* promoter variant + *NPHS1* coding mutation	Easily missed by conventional genetic testing; unresponsive to immunosuppressive therapy, with more severe proteinuria than single coding region mutation

**Table 3:** Immunological subtypes and mechanisms of immune-mediated SRNS.

Subtype	Diagnostic Biomarkers	Key Mechanisms
T-cell mediated	CD4^+^CD25^+^ Treg <5%; IL-17 > 10 pg/mL; T-bet overexpression	Defective Treg suppression enables Th1/Th17 activation and cytokine-mediated podocyte injury
Circulating factor	suPAR > 3 ng/mL (serum), >1.5 ng/mgCr (urine); FSGS factor positivity	suPAR-uPAR interaction activates αVβ3 integrin signaling, disrupting podocyte adhesion
B-cell mediated	CD19^+^ B cells > 15%; anti-podocyte antibodies; IgG > 16 g/L	Autoantibody-mediated complement activation (C1q pathway) causing podocyte damage

**Table 4:** Correlation of genetic etiologies, therapeutic strategies, and response characteristics across SRNS pathogenic mechanisms.

Mechanism Type	Representative Genes	Core Therapeutic Strategy	Therapeutic Response Characteristics
Slit Diaphragm Disruption	*NPHS1*, *NPHS2*	*NPHS1*: early dialysis/renal transplantation (no effective pharmacotherapy); *NPHS2*: trial of CNI (e.g., tacrolimus) combined with low-protein diet.	*NPHS1*: universal non-response to pharmacotherapy; *NPHS2*: about 30–40% short-term response to CNI, with long-term drug resistance.
Podocyte Cytoskeleton Disorder	*ACTN4*, *INF2*, *TRPC6*	Symptomatic management (blood pressure control, antiproteinuric therapy); avoid potent immunosuppressants; *TRPC6* variants: trial of calcium channel blockers.	<20% response rate to corticosteroids/CNI; management focuses on disease progression delay; majority require eventual renal transplantation.
Signaling Pathway Abnormality	*WT1*, *LAMB2*, *CD2AP*	*WT1/LAMB2*: Early renal transplantation (multidisciplinary evaluation required for associated malformations); *CD2AP*: Trial of CNI combined with glucocorticoids.	*WT1/LAMB2*: no therapeutic response; *CD2AP*: about 40% response to CNI with high recurrence rate.
Energy Metabolism Disorder	*COQ2*, *PDSS2*	Coenzyme Q10 supplementation (10–30 mg/kg/d) combined with symptomatic supportive care.	Most patients demonstrate proteinuria reduction within 1–3 months of CoQ10 supplementation; partial cases achieve complete remission, preventing end-stage renal disease.

## Data Availability

No new data were created or analyzed in this study.
